# Pre-Exposure Prophylaxis with Vasculotide Enhances Survival and Alleviates Hematopoietic and Gastrointestinal Injury Following Lethal Total Body Irradiation

**DOI:** 10.3390/ijms27042001

**Published:** 2026-02-19

**Authors:** Li Wang, Bin Lin, Min Zhai, Lisa Hull, Asher Rothstein, Katherine S. Cleveland, Hengying Ellery, Wanchang Cui, Mang Xiao, Juliann G. Kiang

**Affiliations:** 1 Armed Forces Radiobiology Research Institute, Uniformed Services University of the Health Sciences, Bethesda, MD 20814, USA; 2 Henry M. Jackson Foundation for the Advancement of Military Medicine, Inc., Bethesda, MD 20817, USA; 3 Department of Pathology, Uniformed Services University of the Health Sciences, Bethesda, MD 20814, USA; 4 Comparative Pathology, Department of Laboratory Animal Resources, Uniformed Services University of the Health Sciences, Bethesda, MD 20814, USA; 5 Department of Pharmacology and Molecular Therapeutics, Uniformed Services University of the Health Sciences, Bethesda, MD 20814, USA; 6 Department of Medicine, Uniformed Services University of the Health Sciences, Bethesda, MD 20814, USA

**Keywords:** acute radiation syndrome, prophylaxis, Vasculotide, Tie2 pathway, vascular endothelium, total body irradiation, 30-day survival, vascular activation and injury, proinflammatory cytokine/chemokine, hematopoietic and gastrointestinal recovery

## Abstract

No US Food and Drug Administration (FDA)-approved prophylaxis is currently available for Acute Radiation Syndrome (ARS), which remains a significant threat to military and civilian populations. In this study, we investigated Vasculotide (VT), a Tie2 receptor agonist mimic, as a novel pre-exposure prophylaxis designed to stabilize the vascular endothelium, one of primary targets of radiation-induced damage. To evaluate its efficacy, female B6D2F1/J mice were exposed to 9.5 Gy total body irradiation (TBI), with VT administered subcutaneously at 12 and 2 h prior to exposure. Assessments included 30-day survival, biomarkers of vascular injury, proinflammatory cytokine/chemokine profiling, and evaluation of hematopoietic (H) and gastrointestinal (GI) recovery. Our findings demonstrate that VT significantly increased 30-day survival in a dose-dependent manner, achieving a 30% survival advantage at the 20 μg/kg dose. Furthermore, VT provided robust protection against radiation-induced vascular activation and injury, effectively alleviating damage to the bone marrow (BM) and GI tract. Taken together, these results identify VT as a promising prophylactic countermeasure for ARS. By targeting the Tie2 pathway to preserve vascular integrity, VT addresses a critical gap in medical countermeasures, offering a viable strategy to enhance survival and accelerate multi-organ recovery in radiological mass-casualty scenarios.

## 1. Introduction

There is a profound and critical gap in medical preparedness: No US FDA-approved radiation prophylaxis that is practical or deployable for use by military forces prior to radiation exposure [1]. The threat of ARS is not limited to nuclear conflict but extends to accidental radiological events, representing a major vulnerability in global security and public health emergency planning [2]. The complex threat of acute ionizing radiation (IR) exposure precipitates severe multi-organ injury [3], which, in the absence of an effective prophylactic agent, significantly jeopardizes warfighter readiness and survivability in radiation-threat environments. The successful development of a pre-exposure prophylaxis (Pre-P) would directly address this strategic and operational capability gap.

When endothelial cells (ECs) are directly injured by radiation, the primary effect is the breakdown of the endothelial barrier [4,5,6]. This leads to increased vascular permeability and the physical leakage of plasma proteins and fluids into the interstitial space, causing tissue edema [4,5,6]. Injured endothelial cells shift from a quiescent, anti-inflammatory state to an activated, pro-inflammatory state [6,7,8,9]. Once the inflammatory cascade is initiated, the recruited immune cells further amplify the damage to the endothelium [8,9]. Recruited leukocytes release massive amounts of highly potent inflammatory mediators, including reactive oxygen species (ROS), proteases, and pro-inflammatory cytokines/chemokines. These mediators are toxic to the surrounding tissue and, crucially, can cause a secondary injury and apoptosis of neighboring ECs [9,10]. In the context of ARS, this uncontrolled cycle is responsible for the systemic shock, multi-organ failure, and overall lethality associated with the GI- and H-ARS [11,12].

The Angiopoietin (Ang)/Tyrosine kinase with immunoglobulin and EGF homology domains 2 (Tie2) receptor system is a central regulator of vascular homeostasis and stabilization [13,14]. This pathway controls key endothelial processes, including barrier function, vascular permeability, and modulation of inflammatory and immune responses [6,13,14]. Dysregulation of Tie2 signaling is a common pathological feature in various vascular diseases, highlighting the pathway’s immense therapeutic potential as a drug target [14,15,16]. Activation of the Tie2 receptor reduces EC apoptosis, enhances their survival, and promotes angiogenesis [17]. Specifically, Tie2 agonists have been shown to drive hem-angiogenic regeneration following myelosuppression and facilitate recovery from radiation-induced BM damage [18,19]. VT, a polyethylene glycol (PEG)-clustered Tie2-agonist tetrameric peptide (HHHRHSF), mimics the native ligand Ang1 by binding and clustering Tie2 receptors, thereby activating downstream signaling [20,21,22,23,24,25,26,27]. Extensive preclinical research demonstrates that VT effectively protects the vasculature by reducing endothelial apoptosis, preventing capillary leakage, and suppressing harmful neutrophil transmigration across various acute vascular disorders [20,21,22,23,24,25,26,27], such as abdominal sepsis [20], hemorrhagic shock [26], acute kidney injury [25], and various pulmonary and neurological disorders [21,23,27]. Although these findings confirm the potent protective and regenerative functions of the Ang1/Tie2 axis, the precise role and efficacy of the targeted Tie2 signaling in preventing acute radiation-induced endothelial dysfunction, particularly in ARS, remains insufficiently understood and requires comprehensive characterization. In this study, we assessed the prophylactic efficacy of VT in improving mouse survival following lethal irradiation. We further evaluated its protective effects against BM and GI injury. Our working hypothesis is that VT ameliorates BM and GI damage, thereby reducing mortality by preventing the EC damage and suppressing inflammation from irradiation.

## 2. Results

### 2.1. VT Administration Significantly Enhances 30-Day Survival After TBI

**Figure 1:**
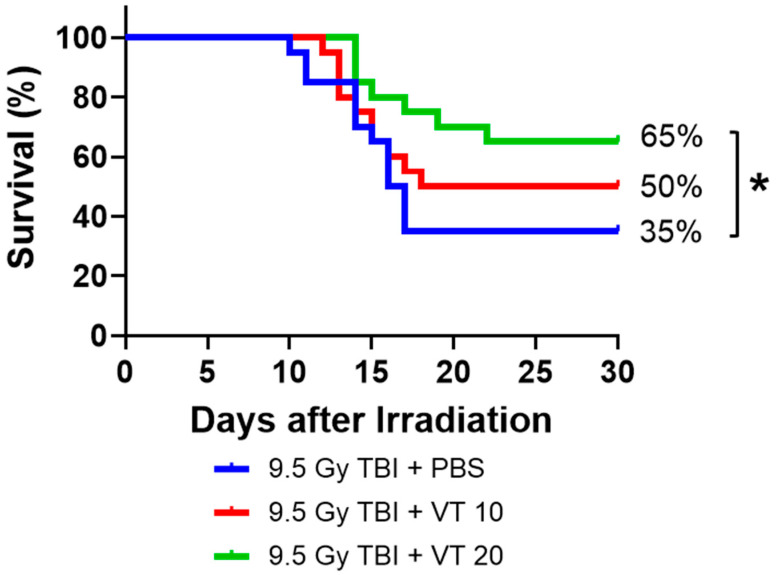
Enhanced 30-day survival by VT administration. Kaplan–Meier survival curves demonstrating the dose-dependent radioprotective effect of VT following lethal TBI, 9.5 Gy. Mice were treated with Vehicle (PBS, blue line), 10 µg/kg VT (red line), or 20 µg/kg VT (green line). The control group (TBI + PBS) resulted in 35% 30-day survival. Treatment with 10 µg/kg VT improved the 30-day survival rate to 50%, and 20 µg/kg VT significantly increased survival to 65%. A comparison between the TBI + PBS and TBI + VT 20 µg/kg groups showed a statistically significant difference (* indicates *p* < 0.05 by Log rank test). n = 20 per group.

We evaluated the radioprotective efficacy of VT on 30-day survival in mice following a lethal dose of 9.5 Gy TBI. In the vehicle control group (TBI + PBS), the 30-day survival rate was 35% (Figure 1), with a median survival time (MeST) of 16.5 days, a mean survival time (MST) of 14.46 days, and a Survival Duration (SD) of 19.0 days (Table 1). VT administration demonstrated a clear dose-dependent protective effect. Treatment with 10 µg/kg VT increased the 30-day survival rate to 50% and extended the MeST to 24.0 days, the MST to 14.60 days, and the SD to 22.30 days. The 20 µg/kg VT dose provided superior protection, achieving a 30-day survival rate of 65%, a 30% increase compared to the control group (Figure 1). This improvement was statistically significant (*p* < 0.05). Notably, the MeST for the 20 µg/kg VT group was >30 days, indicating that more than half of the animals survived through the full observation period. Furthermore, this higher dose improved the MST to 16.43 days and increased the overall survival duration (SD) to 25.25 days (Table 1).

### 2.2. VT Administration Provides Robust Protection Against Radiation-Induced Vascular Activation and Injury

**Figure 2:**
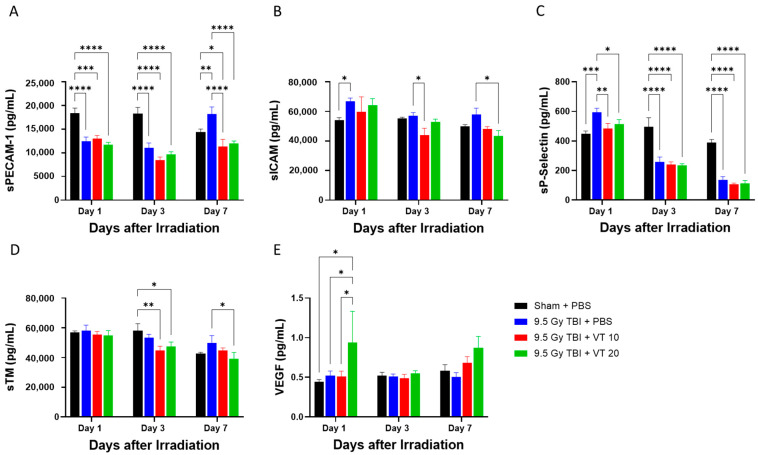
Protection against radiation-induced vascular activation and injury by VT administration. Time course analysis of soluble serum markers for endothelial activation measured on Days 1, 3, and 7 following 9.5 Gy TBI. The experimental groups are Sham + PBS (black bars), 9.5 Gy TBI + PBS (blue bars), 9.5 Gy TBI + VT 10 µg/kg (red bars), and 9.5 Gy TBI + VT 20 µg/kg (green bars). Panels display the concentration (pg/mL) of: (**A**) sPECAM-1, (**B**) sICAM-1, (**C**) sP-Selectin, (**D**) sTM, and (**E**) VEGF. Data are presented as mean ± SEM (n = 5 per group per time point). Statistical analysis was performed using one-way ANOVA followed by Fisher’s LSD multiple comparisons. Statistical significance is indicated by asterisks: * *p* < 0.05, ** *p* < 0.01, *** *p* < 0.001, and **** *p* < 0.0001.

The lethal TBI-induced vascular perturbation was evidenced by significant alterations in circulating adhesion and injury markers (Figure 2). As shown in Figure 2A, TBI caused a significant decrease in soluble Platelet Endothelial Cell Adhesion Molecule-1 (sPECAM-1) levels on Days 1 and 3 relative to Sham controls. While we acknowledge that absolute sPECAM-1 levels in the TBI + PBS group on Day 7 appear numerically similar to Sham levels at earlier time points, this marker is still considered a specific and interpretable indicator of radiation-induced endothelial injury. The biological significance of a biomarker is most accurately interpreted through comparison with concurrent, time-matched controls; this approach effectively isolates radiation-induced deviations from time-related or procedural shifts in the physiological baseline. As shown in Figure 2A, the Sham group exhibits a downward trend in sPECAM-1 from Day 1 to Day 7. In contrast, the TBI + PBS group shows an upward trajectory over the same period. On Day 7, sPECAM-1 levels in the TBI group are significantly elevated compared to the Sham baseline (*p* < 0.01), indicating a clear radiation-induced deviation from the sham control. This “rebound” effect in the TBI group, occurring while Sham levels are declining, demonstrates this marker’s specificity for the radiation-induced vascular perturbation. The validity of sPECAM-1 as a surrogate marker is further reinforced by its response to treatment. Administration of VT (20 µg/kg) significantly suppressed the increase on Day 7 (*p* < 0.0001) and restored sPECAM-1 levels to the range of concurrent Sham control.

Similar trends were observed in other markers of endothelial activation. The TBI + PBS group exhibited elevated soluble Intercellular Adhesion Molecule-1 (sICAM-1) concentrations on Day 1; notably, 20 µg/kg VT significantly reduced sICAM-1 levels by Day 7 compared to the vehicle-treated TBI group (Figure 2B). Radiation also induced a significant increase in soluble Platelet Selectin (sP-Selectin) on Day 1, which was robustly inhibited by both 10 and 20 µg/kg VT (Figure 2C). Interestingly, while TBI subsequently caused a decline in sP-Selectin levels on Days 3 and 7, VT treatment stabilized these fluctuations. In contrast, TBI did not significantly alter soluble Thrombomodulin (sTM; Figure 2D) or Vascular Endothelial Growth Factor (VEGF; Figure 2E) levels compared to Sham controls at the measured time points. However, 20 µg/kg VT treatment significantly reduced sTM on Day 7 and increased VEGF on Day 1 relative to the TBI + PBS group. Collectively, these data indicate that TBI triggers a complex pattern of vascular marker shedding and depletion, which is consistently mitigated by VT administration, suggesting a preservation of endothelial stability.

### 2.3. VT Administration Promotes Hematopoietic Recovery After TBI

**Figure 3:**
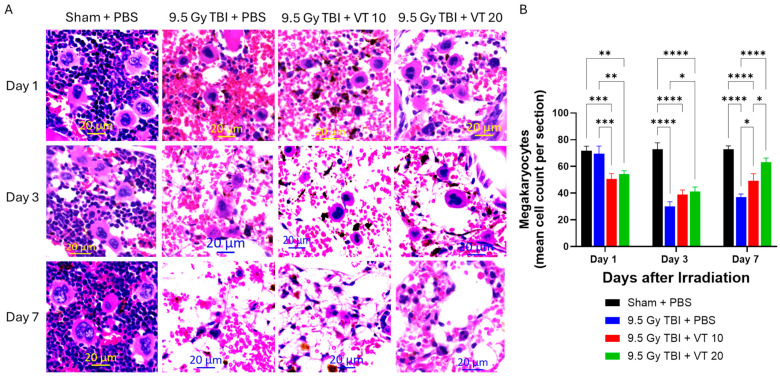
Enhanced hematopoietic recovery and megakaryocyte preservation after TBI by VT administration. Time course analysis of BM morphological damage and repair measured on Days 1, 3, and 7 following TBI at 9.5 Gy. The experimental groups are Sham + PBS (black bars), 9.5 Gy TBI + PBS (blue bars), 9.5 Gy TBI + VT 10 µg/kg (red bars), and 9.5 Gy TBI + VT 20 µg/kg (green bars). Panel (**A**): Representative photomicrographs show H&E staining of sternum sections taken at Days 1, 3, and 7 post-TBI. (Scale bar = 20 µm). Panel (**B**): Quantification of Megakaryocytes (mean cell count per sternum section). Data are presented as mean ± SEM (n = 5 per group per time point). Statistical analysis was performed using one-way ANOVA followed by Fisher’s LSD multiple comparisons. Statistical significance is indicated by asterisks: * *p* < 0.05, ** *p* < 0.01, *** *p* < 0.001, and **** *p* < 0.0001.

The severity of TBI-induced BM damage and the protective efficacy of VT were evaluated via histological analysis of Hematoxylin and Eosin (H&E)-stained sternum sections and the quantification of megakaryocytes (Figure 3). In the Sham control group, the BM maintained high cellularity and an abundance of large, multi-nucleated megakaryocytes throughout the study period (Figure 3A). In contrast, the TBI vehicle control group exhibited rapid and profound BM aplasia. By Days 3 and 7, the hematopoietic compartments were largely replaced by acellular, fatty marrow and sinusoidal congestion, characteristic of a severe H-ARS (Figure 3A). However, both VT treatment groups, particularly the 20 µg/kg, demonstrated a marked preservation of BM cellularity. On Day 7, sternum sections from VT-treated mice showed significant hematopoietic regeneration and increased cellular density.

Quantitative analysis of megakaryocytes mirrored these histological observations (Figure 3B). Following TBI, megakaryocyte counts in the PBS control group declined sharply on Days 3 and 7 relative to Sham controls. VT administration significantly alleviated this depletion; by Day 7, VT at both doses maintained significantly higher megakaryocyte densities compared to the TBI + PBS group (Figure 3B).

### 2.4. VT Administration Accelerates Splenic Recovery After TBI

**Figure 4:**
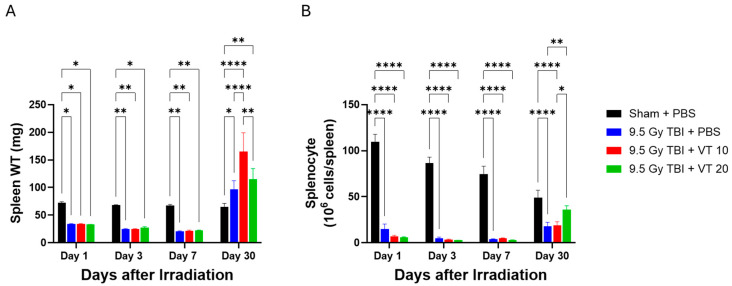
VT administration accelerates splenic recovery following TBI. Time course analysis of splenic injury parameters measured on Days 1, 3, 7, and 30 post-TBI at 9.5 Gy. The experimental groups are Sham + PBS (black bars), 9.5 Gy TBI + PBS (blue bars), 9.5 Gy TBI + VT 10 µg/kg (red bars), and 9.5 Gy TBI + VT 20 µg/kg (green bars). Panels display: (**A**) Spleen WT (in mg), and (**B**) Total Splenocyte Count (in 10^6^ cells per spleen). Data are presented as mean ± SEM. For all groups, n = 5, except for Day 3_9.5 Gy TBI + VT 20 group, where n = 4 due to sample loss during processing. Statistical analysis was performed using one-way ANOVA followed by Fisher’s LSD multiple comparisons. Statistical significance is indicated by asterisks: * *p* < 0.05, ** *p* < 0.01, , and **** *p* < 0.0001.

Lethal TBI resulted in acute and severe splenic injury, characterized by rapid organ atrophy and profound cellular depletion (Figure 4). In the vehicle-pretreated TBI group (TBI + PBS), spleen weights (WT, Figure 4A) decreased significantly compared to Sham controls on Day 1 and remained severely suppressed through Day 7. Correspondingly, total splenocyte counts (Figure 4B) declined acutely on Day 1 and remained at negligible levels through the first week post-irradiation, indicating nearly total lymphoid and myeloid depletion within the splenic parenchyma. By Day 30, a clear regenerative trend was observed in surviving animals. The VT 20 µg/kg group exhibited a statistically significant increase in total splenocyte counts compared to both the TBI + PBS and TBI + VT 10 µg/kg groups, demonstrating a substantial restoration of splenic cellularity. While the VT at 10 µg/kg also provided partial benefit by increasing spleen weight relative to the vehicle control, the higher dose of VT was required for a robust cellular recovery.

### 2.5. VT Administration Accelerates Peripheral Blood Cell Recovery After TBI

**Figure 5:**
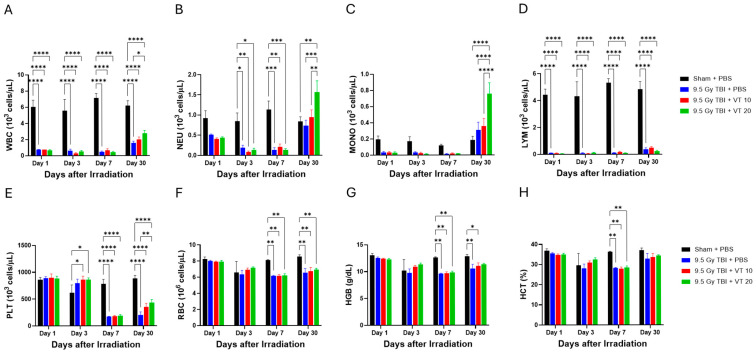
VT administration accelerates peripheral blood cell recovery following TBI. Time course analysis of Complete Blood Counts (CBC) measured on Days 1, 3, 7, and 30 post- TBI at 9.5 Gy. Four experimental groups are shown: Sham + PBS (black bars), 9.5 Gy TBI + PBS (blue bars), 9.5 Gy TBI + VT 10 µg/kg (red bars), and 9.5 Gy TBI + VT 20 µg/kg (green bars). Panels display: (**A**) WBC (10^3^ cells/µL), (**B**) NEU (10^3^ cells/µL), (**C**) MONO (10^3^ cells/µL), (**D**) LYM (10^3^ cells/µL), (**E**) PLT (10^3^ cells/µL), (**F**) RBC (10^6^ cells/µL), (**G**) HGB (g/dL), and (**H**) HCT (%). Data are presented as mean ± SEM. For all groups, n = 5, except for Day 30_9.5 Gy TBI + PBS group, where n = 7, Day 30_9.5 Gy TBI + VT 10 group, where n = 10, Day 30_9.5 Gy TBI + VT 20 group, where n = 10. Statistical analysis was performed using one-way ANOVA followed by Fisher’s LSD multiple comparisons. Statistical significance is indicated by asterisks: * *p* < 0.05, ** *p* < 0.01, *** *p* < 0.001, and **** *p* < 0.0001.

The impact of VT on peripheral blood cell counts was monitored on Days 1, 3, 7, and 30 post-irradiation. Our results reveal that VT administration significantly alleviated the persistent radiation-induced myelosuppression, with the most pronounced benefits observed on day 30 (Figure 5). The vehicle-treated TBI group (TBI + PBS) induced a precipitous decline across all hematopoietic lineages, with total white blood cells (WBC; Figure 5A), neutrophils (NEU; Figure 5B), monocytes (MONO; Figure 5C), and lymphocytes (LYM; Figure 5D) reaching near-zero nadirs on Day 7. VT administration at 20 µg/kg significantly accelerated the reconstitution of myeloid and thrombocytic lineages on Day 30. On Day 30, TBI + VT 20 µg/kg exhibited significantly higher counts of WBCs, NEUs, MONOs, and platelets (PLT; Figure 5E) compared to the vehicle-treated TBI group. In contrast, the recovery of the lymphoid lineage remained severely impaired across all irradiated groups; VT treatment did not yield a statistically significant improvement in lymphocyte populations on Day 30 (Figure 5D). Furthermore, no significant therapeutic benefit was observed in erythroid parameters, including red blood cell counts (RBCs; Figure 5F), hemoglobin (HGB; Figure 5G), and hematocrit (HCT; Figure 5H), throughout the observation period. Collectively, these data indicate that 20 µg/kg VT primarily functions to enhance the recovery of myeloid- and platelet-lineage cells during the acute phase of H-ARS.

### 2.6. VT Administration Elevates Serum Levels of Hematopoietic-Related Cytokines After TBI

**Figure 6:**
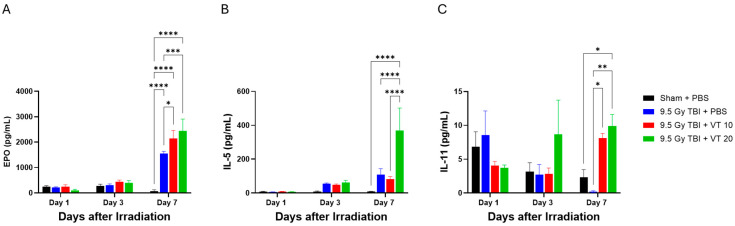
Elevation of serum levels of hematopoietic-related cytokines after 9.5 Gy TBI by VT administration. Time course analysis of key serum cytokines involved in hematopoietic differentiation and growth measured on Days 1, 3, and 7 following 9.5 Gy TBI. The experimental groups are Sham + PBS (black bars), 9.5 Gy TBI + PBS (blue bars), 9.5 Gy TBI + VT 10 µg/kg (red bars), and 9.5 Gy TBI + VT 20 µg/kg (green bars). Panels display the concentration (pg/mL) of: (**A**) EPO, (**B**) IL-5, and (**C**) IL-11. Data are presented as mean ± SEM (n = 5 per group per time point). Statistical analysis was performed using one-way ANOVA followed by Fisher’s LSD multiple comparisons. Statistical significance is indicated by asterisks: * *p* < 0.05, ** *p* < 0.01, *** *p* < 0.001, and **** *p* < 0.0001.

Lethal TBI without or with VT pretreatment significantly modulated the systemic levels of cytokines critical for hematopoietic lineage differentiation and growth (Figure 6). TBI with PBS significantly increased erythropoietin (EPO, Figure 6A) and interleukin-5 (IL-5, Figure 6B), but decreased interleukin-11 (IL-11, Figure 6C) on Day 7, whereas on Day 1 and Day 3 no changes in EPO, IL-5, and IL-11 were observed after TBI. Like the TBI with PBS groups, VT pretreatment at either 10 µg/kg or 20 µg/kg did not alter levels of EPO, IL-5, and IL-11 on both days. However, on Day 7, EPO was further significantly increased by VT at both doses; IL-5 was further significantly increased by VT at 20 µg/kg. Notably, levels of IL-11, a pivotal megakaryocyte growth factor that was markedly suppressed in the TBI + PBS group, were significantly recovered and elevated by VT at both doses on this day.

The rationale for the selection of the subset presented in Figure 6 is as follows: (i) Our primary objective was to identify the signaling pathways that drove the significant recovery observed in the peripheral blood and bone marrow. We prioritized EPO, IL-5, and IL-11 because they were established primary cytokines to act on the erythroid, myeloid/eosinophilic, and megakaryocytic lineages, respectively—the exact compartments where VT demonstrated the most robust rescue (as shown in Figure 3 and Figure 5). (ii) We have also included the complete multiplex dataset (comprising all measured cytokines and chemokines) as Supplementary Tables S1 and S2. By providing the full dataset in the supplement, VT is shown to promote a targeted regenerative signal rather than a non-specific or pro-inflammatory cytokine storm.

### 2.7. VT Administration Enhances GI Recovery After TBI

**Figure 7:**
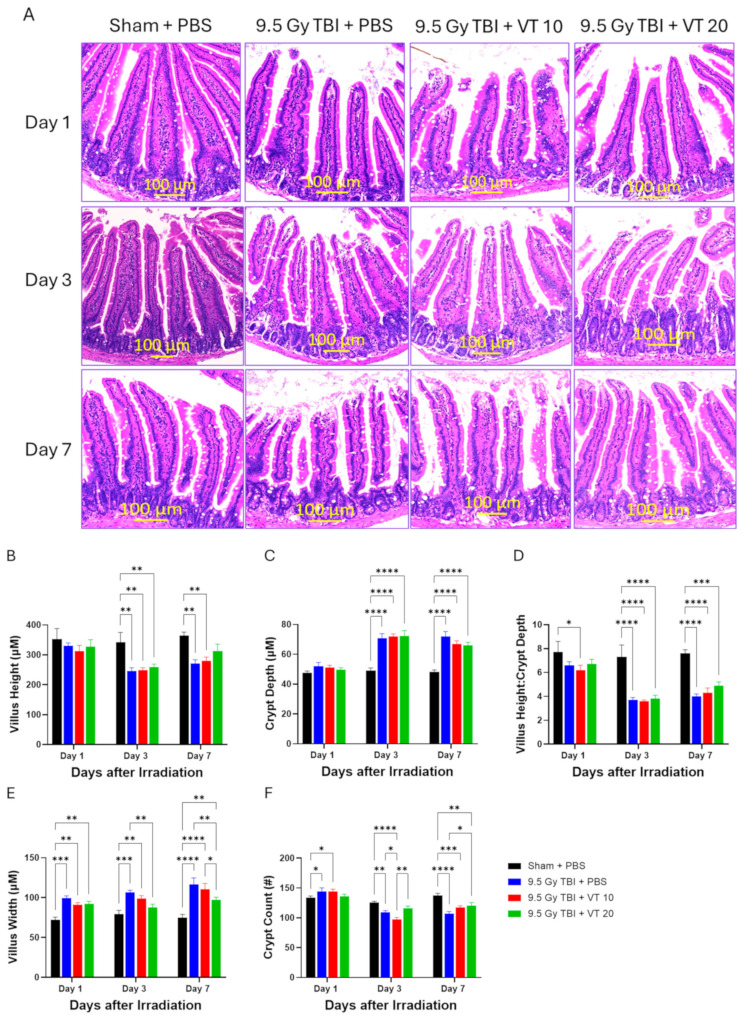
Enhanced GI recovery after TBI by VT pre-administration. Time course analysis of intestinal morphological damage and repair measured on Days 1, 3, and 7 following TBI at 9.5 Gy. The experimental groups are Sham + PBS (black bars), 9.5 Gy TBI + PBS (blue bars), 9.5 Gy TBI + VT 10 µg/kg (red bars), and 9.5 Gy TBI + VT 20 µg/kg (green bars). Panel (**A**): Representative images of H&E-stained small intestine sections (jejunum) taken on Days 1, 3, and 7 post-TBI (100 µm scale bar). Panel (**B**–**F**): Quantification of intestinal parameters, presented as mean ± SEM (n = 5 per group per time point). These panels display (**B**) Villus Height, (**C**) Crypt Depth, (**D**) Villus Height/Crypt Depth Ratio, (**E**) Villus Width, and (**F**) Crypt Count. Statistical analysis was performed using one-way ANOVA followed by Fisher’s LSD multiple comparisons. Statistical significance is indicated by asterisks: * *p* < 0.05, ** *p* < 0.01, *** *p* < 0.001, and **** *p* < 0.0001.

Lethal TBI-induced severe, time-dependent alterations in small intestinal morphology, consistent with GI-ARS (Figure 7). Histological evaluation of the TBI + PBS control group revealed progressive architectural degeneration from Day 1 to Day 7, characterized by marked villus blunting, epithelial swelling, and fusion, alongside profound cellular depletion within the crypts of Lieberkühn (Figure 7A). Pre-administration of 20 µg/kg VT significantly attenuated this radiation-induced mucosal damage and promoted morphological recovery. Quantitative analysis confirmed that while villus width, a marker of inflammatory swelling and structural distortion, was severely increased in the TBI + PBS group through Day 7, VT treatment significantly preserved villus architecture on Days 3 and 7 relative to the vehicle control (Figure 7E). Furthermore, TBI caused a significant reduction in crypt density on Days 3 and 7. However, VT 20 µg/kg significantly enhanced the crypt survival and regenerative activity on Day 7 compared to the TBI + PBS group (Figure 7F). These data suggest that VT provides a protective environment that facilitates the preservation and regeneration of the intestinal stem cell niche.

## 3. Discussion

Our findings demonstrate that VT serves as a potent radioprotector by significantly enhancing survival following lethal TBI in a dose-dependent manner. The superior efficacy of the 20 µg/kg dose suggests a robust therapeutic window for protecting against the radiation-induced injury. We propose that the mechanism of action (MOA) for VT-mediated radioprotection is primarily driven by the modulation of the endothelial response, thereby preserving vascular integrity. VT is administered only prior to irradiation, yet recovery effects are reported on Day 30. The recovery observed on Day 30 is not attributed to the sustained systemic presence of the drug itself (the reported VT half-life is approximately 24 h [20]), but rather to the preservation of regenerative capability during the critical 24–72 h window post-irradiation. The “legacy effect” begins with the immediate reduction in radiation-induced vascular perturbation. By administering VT prior to irradiation, the agent is available to suppress acute endothelial activation (evidenced by the significant reduction in sP-Selectin on Day 1) and induce pro-survival signals like VEGF on Day 1. This prevented the initial “vascular collapse” that would otherwise render the microenvironment hostile to surviving stem cells. Although the dosing was transient, VT triggered a sustained endogenous cytokine response. The significant elevation of EPO, IL-5, and IL-11 observed on Day 7, long after the drug had been metabolized, indicates that VT shifted the systemic environment from a pro-inflammatory state to a pro-regenerative one. These growth factors are known to be responsible for the hematopoietic recovery observed by Day 30 [28]. Our results demonstrate that VT may maintain the physical architecture of the intestinal crypts during the acute phase. By preserving the regenerative capacity for these anatomical niches early on, VT ensures that the surviving fraction of ISCs has a viable scaffold for the subsequent expansion.

Following TBI at 9.5 Gy, we observed significant elevations in serum biomarkers associated with vascular activation and endothelial distress. Specifically, the acute increases in sICAM-1 and sP-Selectin on Day 1 signify an early-phase endothelial perturbation. On Day 7, the TBI + PBS group exhibited a marked pathological rise in sPECAM-1 compared to Sham-irradiated mice, a hallmark of progressive endothelial injury. Conversely, VT administration consistently suppressed the accumulation of these markers. Notably, VT significantly attenuated the elevation of sPECAM-1 and sICAM-1 on Day 7 and blunted the acute sP-Selectin surge on Day 1. Furthermore, VT at 20 µg/kg significantly reduced sTM levels on Day 7 relative to the TBI + PBS group, indicating a reduction in endothelial shedding and membrane degradation. Interestingly, VT at 20 µg/kg induced a transient increase in VEGF on Day 1. Given that VEGF is a critical mediator of angiogenesis and vascular repair, this early induction suggests that VT triggers a pro-regenerative signaling cascade that may accelerate vascular recovery following IR. Our data supports an endothelial protective mechanism for the therapeutic efficacy of VT. On Day 7, sPECAM-1 levels in the VT-treated group (20 µg/kg) were not merely attenuated, compared to the TBI + PBS group, but were statistically indistinguishable from the Sham + PBS group. This normalization to the physiological baseline suggests that VT facilitates a restoration of vascular homeostasis during the subacute phase of injury. In addition, VT treatment (20 µg/kg) triggered a significant increase in VEGF levels on Day 1. As VEGF is a critical mediator of endothelial repair and survival, this early pro-survival spike indicates that VT actively bolsters the vasculature’s resilience against radiation. If VT were simply a non-specific inhibitor of marker shedding, we would not expect to see this targeted upregulation of regenerative growth factors.

Furthermore, the reduction in sPECAM-1 is not an isolated finding; it correlates strictly with the preservation of the vascular niche in the 9.5 Gy TBI + VT at 20 µg/kg group. In the context of lethal IR, the parenchymal tissue recovery is functionally dependent on the vascular restoration. Within the BM, hematopoietic stem cells (HSCs) reside in close proximity to sinusoidal ECs; the collapse of these microvessels leads to the loss of the hematopoietic niche that results in the subsequent BM failure [12]. Beyond the structural support, healthy ECs actively facilitate DNA repair in irradiated stem cells [29] and release pro-regenerative angiocrine factors, such as VEGF and Ang-1, which trigger the proliferation of surviving stem cell pockets [30].

Our study demonstrates that VT administration, particularly at 20 µg/kg, significantly preserves BM cellularity. On Day 7, VT-treated mice exhibited distinct histological signs of active hematopoiesis and increased cellular density, contrasting with the severe marrow aplasia observed in the TBI control group. This preservation of the BM microenvironment correlates with the robust recovery of peripheral blood counts observed on Day 30. Furthermore, VT pretreatment significantly alleviated megakaryocyte depletion. On Day 7, the VT 20 µg/kg group maintained significantly higher megakaryocyte counts compared to TBI + PBS controls, which directly paralleled the enhanced platelet count recovery observed in the peripheral blood on Day 30. These downstream biological effects confirm that the lower sPECAM-1 levels reflect a preserved endothelial environment rather than an isolated marker suppression.

We further evaluated serum levels of key hematopoietic cytokines following Gy TBI at 9.5. In the TBI + PBS group, IL-11, a critical driver of megakaryocytic lineage regeneration, dropped to near-baseline levels by Day 7, signaling a collapse of the platelet-regulatory system. VT administration (10 and 20 µg/kg) successfully prevented this decline, maintaining significantly higher IL-11 levels during the subacute phase (Day 7). This sustained IL-11 expression likely provides essential signaling stimuli required for the long-term platelet count recovery noted on Day 30. While initial cellular loss is similar between PBS-treated and VT-treated TBI groups, the “protective” nature of VT is defined by its ability to prevent the subsequent escalation of damage. The benefit of VT is mostly evident in the divergence of the two groups after Day 3. Although bone marrow cellularity dropped initially in both, VT-treated groups maintained a “competent” microenvironment. This was supported by the preservation of bone marrow architecture and IL-11/EPO levels, which prevented the transition from acute injury to irreversible marrow aplasia and fatty cell replacement. In a 9.5 Gy TBI model, total prevention of initial damage is biologically unlikely. Therefore, we define the VT’s effect as offering a successful shielding of the underlying tissue “infrastructure.” By buffering the system against the subsequent “vascular breakdown” and cytokine storm, VT preserves a regenerative window that is absent in the TBI group.

Interestingly, while we observed significant differences in NEU and MONO counts between groups, there were no significant changes in Granulocyte Colony-Stimulating Factor (G-CSF), Granulocyte-Macrophage Colony-Stimulating Factor (GM-CSF), Interferon-gamma (IFNγ), and Interleukin-3 (IL-3) levels (Supplemental Table S1), suggesting that the VT-mediated recovery of the myeloid lineage may not be driven by a systemic surge in these colony-stimulating factors. Instead, the accelerated recovery of NEU and MONO populations may result from localized paracrine signaling within the preserved vascular niche or an increase in the sensitivity of myeloid progenitors to baseline cytokine levels, rather than an increase in the systemic cytokine concentration.

High-turnover tissues, most notably the GI epithelium, are fundamentally dependent on a specialized microenvironmental niche for homeostatic maintenance and post-injury regeneration. The intestinal stem cells (ISCs) residing at the base of the crypts of Lieberkühn are supported by an intricate subepithelial capillary network. The radiation-induced vascular rarefaction, the progressive loss of microvessels, results in localized ischemia and nutrient deprivation within these crypts, ultimately precipitating mucosal barrier breakdown and the onset of GI syndrome [31].

Our findings demonstrate that VT administration, particularly at the 20 µg/kg dose, confers a dual protective benefit by preserving both the absorptive villi and the regenerative crypt compartments of the small intestine. This structural preservation is most pronounced during the subacute phase on Day 7 post-IR. This timeframe represents a critical clinical window in GI-ARS; it is the period during which denudation of the intestinal mucosa typically leads to bacterial translocation, systemic sepsis, and multi-organ failure. By maintaining the integrity of the crypt-villus axis during this vulnerable period, VT appears to shorten the gap between acute injury and successful epithelial reconstitution, thereby significantly reducing the radiation-induced mortality.

While HSC loss is the primary insult in H-ARS, our data underscore that the survival and expansion of the surviving stem cell fraction depend on a functional vascular niche. The preservation of bone marrow cellularity and megakaryocytes in VT-treated mice (Figure 6) suggests that VT stabilizes the structural and signaling environment necessary for endogenous HSCs to rebuild the marrow. Similarly, while Intestinal stem cells (ISCs) are the primary drivers of intestinal repair, their survival is linked to the health of the underlying microvasculature. Our findings show that VT (20 µg/kg) significantly enhances crypt count recovery. We interpret this not as VT creating new stem cells, but as VT alleviating the ischemia and/or inflammatory signaling that typically exacerbates ISC loss. Therefore, by suppressing markers of vascular activation (sPECAM-1, sP-Selectin) and elevating pro-regenerative cytokines (VEGF, EPO, IL-11), VT optimizes the systemic environment to support the surviving stem cells to initiate tissue repair. Our data confirms that while 10 µg/kg provided partial protection, the 20 µg/kg dose was required to achieve statistically significant multi-organ recovery. Specifically, only the 20 µg/kg cohort significantly achieved 30-day survival (Figure 1), restoration of splenocyte counts (Figure 4B), comprehensive peripheral blood recovery (Figure 5), and GI recovery (Figure 7E,F).

The identification of VT as a potent radioprotector is particularly significant in viewing the current landscape of MCMs. As detailed in recent comprehensive reviews of the field, most established MCMs primarily target lineage-specific hematopoietic recovery or organ-specific recovery [1]. We have noted that Romiplostim can achieve high rescue rates and multiple organ injury mitigation [32,33]. By robustly stabilizing the megakaryocyte/platelet lineage, Romiplostim may alleviate early hemorrhage and systemic inflammation, thereby facilitating an environment conducive to endogenous recovery in other tissues [32,33]. While Romiplostim acts as a potent, targeted agonist for the TPO receptor, our data suggests VT functions as a biological orchestrator. VT administration triggers a synchronized surge in a cocktail of endogenous cytokines, including EPO (erythroid), IL-5 (myeloid), and IL-11 (megakaryocytic/mucosal). This broader cytokine profile may offer a unique advantage in responding to the heterogenous cellular damage induced by a high-dose TBI. A distinguishing feature of VT is its simultaneous modulation of the vascular endothelial infrastructure. As a result, suppressing vascular activation and preserving the physical integrity of the splenic and intestinal niches by VT pretreatment suggest that the vascular component of ARS is one of the critical targets.

In summary, our study identifies VT as a highly effective, dose-dependent radioprotector that alleviates the lethal effects of TBI. The primary mechanism of VT-mediated survival appears to be a preservation and rapid stabilization of the microvascular endothelium. This vascular preservation provides an essential scaffold and paracrine signaling environment necessary for the regeneration of hematopoietic stem cells and GI repair.

While we observed changes in serum biomarkers like sPECAM-1 and IL-11, the precise intracellular signaling pathways (e.g., Tie2, Akt, or Notch signaling) through which VT stabilizes the endothelium and promotes tissue regeneration warrant further investigation. Long-term studies are also needed to determine if VT acute vascular protection translates to a reduction in delayed effects of acute radiation exposure (DEARE), such as the radiation-induced fibrosis or cardiovascular complications.

## 4. Materials and Methods

### 4.1. Animals

B6D2F1/J female mice, at 14 weeks of age at the time of irradiation, were obtained from the pathogen- and opportunist-free maximum barrier facility at The Jackson Laboratory (Bar Harbor, ME, USA). Mice were housed in an immunocompromised-animal room within an Association for Assessment and Accreditation of Laboratory Animal Care (AAALAC)-accredited facility managed by the Department of Laboratory Animal Resources (DLAR) at the Uniformed Services University of the Health Sciences (USUHS). Upon arrival, all animals were acclimated 3 weeks prior to the start of the study. All animal study procedures were performed in strict adherence to the protocols approved by the USUHS Institutional Animal Care and Use Committee (IACUC), and in accordance with DLAR guidelines and federal regulations [34].

### 4.2. VT Administration

VT (Cat No. CRB1001060; BioSynth International Inc., Gardner, MA, USA) was reconstituted according to the manufacturer’s specifications and prepared for the final study concentrations using sterile PBS as the vehicle. VT was administered via subcutaneous (SC) injection at the dose of 10 µg/kg or 20 µg/kg (in a volume of 0.2 mL per mouse) at 12 h and 2 h prior to TBI. PBS was administered to control groups using the same route and corresponding volume and schedule. The doses of 10 and 20 µg/kg were selected based on prior preclinical models of sepsis [20], influenza [35], lung injury [21], tumor metastasis [36], and cutaneous radiation injury [37], where these doses demonstrated Tie2 activation and endothelial barrier stabilization.

### 4.3. TBI Procedure

TBI was performed at AFRRI’s ^60^Co γ-irradiation facility, following previously established protocol [34]. Mice were restrained in custom-designed Lucite boxes, partitioned into eight individual compartments. Animals were irradiated simultaneously in a bilateral field configuration. The radiation (9.5 Gy) was delivered at an estimated dose rate of ∼0.4 Gy/min. Immediately post-irradiation, animals were returned to their home cages [34].

### 4.4. Experimental Design

The experimental design is summarized in Table 2 and Table 3. A total of 125 mice were utilized for this study. The sample size of n = 20 mice per group for 30-day survival study and n = 5 mice for mechanistic study is based on Power analysis. Randomization by body weight balancing was used to allocate animals to experimental groups. The spatial arrangement of cages was identical for all three irradiated groups to minimize environmental bias. Investigators were aware of the group allocation at the different stages of the experiment during the allocation, the conduct of the experiment, the outcome assessment, and the data analysis.

### 4.5. Survival Study

Survival was tracked daily for 30 days post-TBI. All animals were monitored a minimum of twice daily throughout the entire study duration, guided by the Institutional IACUC Rodent Intervention Score Sheet. During the periods of highest expected mortality (Day 10–20 post-TBI), monitoring frequency was increased to three times daily (at least 6 h apart) to ensure timely intervention. Mice were humanely euthanized immediately upon exhibiting signs of moribundity (e.g., significant weight loss, lethargy, persistent neurological deficits, or inability to stand or right themselves). Specific predetermined parameters include: (i) Loss of >35% of baseline body weight; (ii) Failure to exhibit a righting reflex (inability to stand or right themselves within 5 s); (iii) Signs of respiratory distress (labored breathing). Euthanasia Method: Euthanasia was performed via CO_2_ inhalation followed by cervical dislocation as a secondary confirmatory method, in strict accordance with the institutional IACUC guidelines [34].

### 4.6. Blood Collection and Serum Preparation

At pre-determined time points, such as days 1, 3, and 7 for mechanistical evaluation and day 30 for survival study, mice were anesthetized under isoflurane for terminal blood collection via cardiac puncture. Whole blood was partitioned into two aliquots: 15–30 µL was collected into K_2_EDTA-coated microtubes (REF365974, BD, Franklin Lakes, NJ, USA) for CBC analysis, while the remaining volume was transferred to serum separator tubes (REF365967, BD, Franklin Lakes, NJ, USA). For serum preparation, the blood was allowed to clot undisturbed at room temperature for 30–60 min. Following clot formation, samples were centrifuged at 10,000× *g* for 10 min at 4 °C. The resulting supernatant (serum) was carefully aspirated, partitioned into aliquots to prevent repeated freeze–thaw cycles, and stored at −80 °C for downstream analysis [34].

### 4.7. Tissue Collection

Immediately following terminal blood collection, confirmatory cervical dislocation was performed. The sternum, spleen, and jejunum were harvested for analysis. The sternum and a segment of the jejunum were promptly fixed in 10% neutral buffered formalin for subsequent histological evaluation. The spleen was excised and placed into 12-well plates containing ice-cold PBS for the determination of spleen weight and total splenocyte quantification [34].

### 4.8. CBC

Whole blood samples collected in K_2_EDTA-coated microtubes were analyzed for CBC with differential. Automated analysis was performed using the Element HT5 veterinary hematology analyzer (Heska, Loveland, CO, USA) in accordance with the manufacturer’s instructions [38].

### 4.9. Inflammatory Cytokines/Chemokines

Inflammatory cytokines/chemokines were measured in serum by Eve Technologies (Calgary, AB, Canada). We measured the following 44 cytokines and chemokines: eotaxin, G-CSF, GM-CSF, IFNγ, IL-1α, IL-1β, IL-2, IL-3, IL-4, IL-5, IL-6, IL-7, IL-9, IL-10, IL-12p40, IL-12p70, IL-13, IL-15, IL-17, IP-10, KC, LIF, LIX, M-CSF, MCP-1, MIG, MIP-1α, MIP-1β, MIP-2, RANTES, TNFα, VEGF-A, 6Ckine/Exodus 2, erythropoietin, fractalkine, IFNβ-1, IL-11, IL-16, IL-20, MCP-5, MDC, MIP-3α, MIP-3β, and TARC [39].

### 4.10. Biomarkers for Vascular Injury

Biomarkers for vascular pathology were measured in serum by Eve Technologies (Calgary, AB, Canada). We measured the following panel: ProMMP-9, PAI-1 (Total), sPECAM-1, sP-Selectin, sE-Selectin, sICAM-1, and sThrombomodulin [38].

### 4.11. Histological Examination of the Sternum and Jejunum

Harvested sternum and proximal jejunum tissues were immediately fixed in 10% neutral buffered formalin for 24 h, rinsed with PBS three times, and transferred to 70% ethanol for storage. Sternum samples underwent an additional decalcification step prior to further processing. All tissues were then dehydrated, embedded in paraffin, and sectioned at a thickness of 5 μm onto charged glass slides. Following H&E staining, bright-field images were acquired using a Zeiss Axio Scan.Z1 slide scanner. Image analysis and processing were performed using Zeiss ZEN 2.5 (blue edition; Carl Zeiss AG, Oberkochen, Germany), and Fiji-windows-x64 software (National Institutes of Health, Bethesda, MD, USA) [34].

Quantification of megakaryocytes: While steady-state megakaryocytes are defined by exceptionally large size, abundant eosinophilic cytoplasm, and multi-lobulated nuclei, regenerative megakaryocytes following radiation often exhibit distinct morphological shifts, including smaller overall cell size and nuclear hypolobulation (single-lobed or round nuclei). During the active repair phase post-irradiation, the bone marrow microenvironment prioritizes the expansion of the megakaryocyte-erythroid progenitors (MEPs) compartment [40]. These progenitors facilitate the rapid lineage-specific reconstitution required to restore systemic hematopoietic homeostasis [41]. Quantification was performed by an experienced investigator, counting total megakaryocytes (both steady-state and regenerative morphologies) across three sternebral segments per animal at 400× magnification. Data are expressed as the mean number of megakaryocytes per sternebral segment.

### 4.12. Spleen Weight and Splenocyte Quantification

Excised spleens were weighed and immediately placed in plastic pouches containing 10 mL of Hanks’ Balanced Salt Solution (HBSS; Gibco; Life Technologies Inc., Carlsbad, CA, USA). The tissue was homogenized using a Stomacher^®^ 80 Biomaster Lab System (Seward Laboratory Systems, Port St. Lucie, FL, USA) at a high speed for 60 s. The resulting homogenate was passed through a 70-μm cell strainer (Falcon™, Durham, NC, USA) and centrifuged at 1960× *g* for 10 min. To remove residual red blood cells, the cell pellet was resuspended in 10 mL of erythrocyte lysis buffer (Qiagen, Hilden, Germany) and incubated at 37 °C for 10 min. Following lysis, the splenocytes were pelleted at 1960× *g* for 10 min and resuspended in 10 mL of PBS. Total splenocyte counts were then determined using a Countess™ automated cell counter (Invitrogen™, Carlsbad, CA, USA) [34].

### 4.13. Statistical Analysis

To ensure comprehensive analysis, no exclusion criteria were established; all animals and data points were included in the final data set. Statistical analyses were conducted using GraphPad Prism version 10 (GraphPad Software, La Jolla, CA, USA). Data are presented as mean ± standard error of the mean (SEM). Survival data were analyzed using Kaplan–Meier curves, and differences between groups were evaluated using the log-rank (Mantel–Cox) test. For comparisons among multiple experimental groups, one-way or two-way analysis of variance (ANOVA) was performed, followed by Fisher’s LSD post hoc test for multiple comparisons. For comparisons between two experimental groups, unpaired *t*-test with Welch’s correction was performed. Statistical significance was defined as *p* < 0.05.

## Figures and Tables

**Table 1:** Primary outcome in a 30-day survival study.

Group	Median Survival Time (MeST, Days) ^1^	Mean Survival Time (MST, Days) ^2^	Survival Duration (SD, Days) ^3^
9.5 Gy TBI + PBS	16.5	14.46	19.0
9.5 Gy TBI + VT 10 ^4^	24.0	14.60	22.30
9.5 Gy TBI + VT 20 ^5^	>30.0	16.43	25.25 *

^1^ Median Survival Time: the time point at which 50% of the mice in this study were still alive. ^2^ Mean Survival Time: the average of the time points at which each animal in this study either died or met a predetermined humane endpoint. ^3^ Survival Duration: the average overall length of time the mice remain alive in the study. ^4^ VT 10: Vasculotide at 10 µg/kg. ^5^ VT 20: Vasculotide at 20 µg/kg. * *p* < 0.05 for 9.5 Gy TBI + PBS vs. 9.5 Gy TBI + VT 20 by unpaired t test with Welch’s correction.

**Table 2:** Experimental design for a 30-day survival study.

Group	PBS	VT (10 µg/kg)	VT (20 µg/kg)
Sham ^1^	n = 5	n = 0	n = 0
9.5 Gy TBI	n = 20	n = 20	n = 20
Total			n = 65

^1^ Survival rates were analyzed only for the 9.5 Gy TBI groups. The Sham + PBS group provided baseline control data for spleen weight, splenocyte density, and CBC parameters.

**Table 3:** Experimental design for mechanistic study.

Group	PBS	VT (10 µg/kg)	VT (20 µg/kg)
Sham	n = 15 ^1^	n = 0	n = 0
9.5 Gy TBI	n = 15 ^1^	n = 15 ^1^	n = 15 ^1^
Total			n = 60

^1^ On Days 1, 3, and 7 post-TBI, five mice per group were euthanized for blood and tissue collection.

## Data Availability

The multiplex dataset is included in the Supplementary Material. Other data presented in this study are available from the corresponding authors upon request.

## Supplementary Materials

The following supporting information can be downloaded at: https://www.mdpi.com/article/10.3390/ijms27042001/s1.

## Abbreviations

The following abbreviations are used in this manuscript:

AAALAC	Association for Assessment and Accreditation of Laboratory Animal Care
Ang	Angiopoietin
ARS	Acute radiation syndrome
BM	Bone marrow
DEARE	Delayed effects of acute radiation exposure
DLAR	Department of Laboratory Animal Resources
ECs	Endothelial cells
EPO	Erythropoietin
FDA	Food and Drug Administration
G-CSF	Granulocyte Colony-Stimulating Factor
GI	Gastrointestinal
GM-CSF	Granulocyte-Macrophage Colony-Stimulating Factor
H	Hematopoietic
H&E	Hematoxylin and Eosin
HBSS	Hanks’ balanced salt solution
HCT	Hematocrit
HGB	Hemoglobin
HJF	Henry M. Jackson Foundation for the Advancement of Military Medicine, Inc.
HSCs	Hematopoietic stem cells
IAA	Interagency agreement
IACUC	Institutional animal care and use committee
IFNγ	Interferon-gamma
IL-11	Interleukin-11
IL-3	Interleukin-3
IL-5	Interleukin-5
IR	Ionizing radiation
ISCs	Intestinal stem cells
LYM	Lymphocytes
MeST	Median Survival Time
MOA	Mechanism of action
MONO	Monocytes
MST	Mean Survival Time
NEU	Neutrophils
PEG	Polyethylene glycol
PLT	Platelets
Pre-P	Pre-exposure prophylaxis
RBC	Red blood cell
ROS	Reactive oxygen species
SC	Subcutaneous
SD	Survival duration
SEM	Standard error of the mean
sICAM-1	Soluble intercellular adhesion molecule-1
sPECAM-1	Soluble platelet endothelial cell adhesion molecule-1
sP-Selectin	Soluble platelet selectin
sTM	Soluble thrombomodulin
TBI	Total body irradiation
Tie2	Tyrosine kinase with immunoglobulin and EGF homology domains 2
USUHS	Uniformed Services University of the Health Sciences
VEGF	Vascular Endothelial Growth Factor
VT	Vasculotide
WBC	White blood cells
WT	Weight
