# Supplementary material for: Pre-Exposure Prophylaxis with Vasculotide Enhances Survival and Alleviates Hematopoietic and Gastrointestinal Injury Following Lethal Total Body Irradiation
Source: Int J Mol Sci. 2026 Feb 19;27(4):2001. doi: 10.3390/ijms27042001 (PMC12941350; doi:10.3390/ijms27042001)
Supplement: Supplementary file 1 [file ijms-27-02001-s001.zip › ijms-4129722 Supplemental Table S1 (final).pdf]

Supplemental Table S1. Multiplex analysis of serum cytokine profiles in mice on Days 1, 3, and 7 post-TBI.

	Day 1				Day 3				Day 7			
Cytokine	S+V	TBI+ V	TBI+ VT10	TBI+ VT20	S+V	TBI+ V	TBI+ VT10	TBI+ VT20	S+V	TBI+ V	TBI+ VT10	TBI+ VT20
G-CSF*	164.50 ±32.53	378.5± 103.8	621.60 ±76.09	465.80 ±35.11	116.40 ±20.60	1500± 167[a]	803.6± 75.3 [b]	1798± 184[d]	75.30± 10.64	1780± 145[a]	2451± 449[b]	1763± 522[d]
GM-CSF	0.00± 0.00	0.06± 0.06	1.19± 0.76	5.32± 5.32	0.79± 0.72	3.44± 1.48	0.00± 0.00	1.94± 1.17	4.19± 4.19	0.00± 0.00	0.47± 0.47	3.47± 1.70
M-CSF	8.07± 1.36	5.76± 1.16	7.61± 1.01	7.93± 0.82	6.95± 1.13	8.06± 1.06	4.76± 0.63[b]	6.95± 1.06	5.77± 1.13	4.97± 0.48	8.82± 0.51[b]	7.42± 1.53
IFNγ	0.07± 0.07	0.00± 0.00	0.68± 0.68	6.25± 5.84[c,d]	0.74± 0.74	0.21± 0.21	0.00± 0.00	0.00± 0.00	1.12± 1.12	0.00± 0.00	3.19± 0.76	0.00± 0.00
IFNβ-1	116.05 ±15.66	90.09± 13.01	71.69± 16.31	73.56± 9.67	80.85± 14.85	95.71± 24.67	69.86± 5.60	773± 696[c,d]	75.52± 3.37	46.38± 11.39	131.61 ±16.27	128.31 ±9.11
IL-1α	27.61± 8.80	25.57± 12.39	57.19± 7.42	45.77± 10.97	50.32± 31.47	95.25± 17.15	75.74± 9.36	125.8± 17.6[d]	30.91± 12.48	94.59± 7.62[a]	108.54 ±20.05	89.07± 19.93
IL-1β	5.50± 3.05	0.62± 0.62	5.84± 3.10	1.34± 1.34	0.62± 0.62	1.96± 1.33	0.62± 0.62	0.00± 0.00	6.16± 5.92	4.78± 3.67	2.00± 2.00	2.00± 2.00
IL-2	0.56± 0.43	0.88± 0.64	0.23± 0.23	6.45± 5.66[c,d]	0.00± 0.00	0.46± 0.28	0.45± 0.45	0.00± 0.00	0.55± 0.55	0.00± 0.00	0.23± 0.23	0.91± 0.42
IL-3	0.48± 0.33	0.49± 0.32	0.54± 0.16	0.80± 0.44	0.32± 0.21	0.46± 0.21	0.36± 0.22	0.50± 0.16	0.33± 0.09	0.83± 0.20	1.12± 0.35	1.17± 0.50
IL-4	0.55± 0.06	0.40± 0.05	0.54± 0.09	0.44± 0.04	0.53± 0.06	0.52± 0.04	0.39± 0.03	0.47± 0.05	0.50± 0.09	0.47± 0.07	0.61± 0.03	0.53± 0.03
IL-5	8.07± 2.27	6.75± 0.60	8.72± 0.97	7.32± 0.71	8.63± 3.54	56.24± 3.14	47.92± 5.34	61.68± 12.94	9.02± 1.16	107.64 ±35.78	81.25± 15.75	368± 134[c,d]
IL-6	7.84± 3.17	5.37± 1.52	10.64± 2.11	6.20± 1.30	1.42± 0.50	10.57± 1.51[a]	5.96± 1.15	13.61± 5.79	1.98± 0.56	6.54± 1.58	12.83± 3.55	12.24± 5.08
IL-7	3.90± 3.90	2.32± 2.32	0.56± 0.56	0.00± 0.00	0.00± 0.00	0.00± 0.00	0.06± 0.06	1.84± 1.27	0.39± 0.39	0.00± 0.00	1.89± 0.73	2.00± 2.00
IL-9	0.00± 0.00	0.00± 0.00	0.00± 0.00	0.00± 0.00	0.00± 0.00	0.00± 0.00	0.00± 0.00	2.78± 2.78	4.58± 3.38	0.00± 0.00[a]	2.78± 2.78	0.00± 0.00
IL-10	3.74± 2.43	0.80± 0.80	1.60± 0.98	0.00± 0.00	1.05± 0.78	0.05± 0.05	0.00± 0.00	5.63± 5.63[c,d]	0.00± 0.00	0.00± 0.00	8.75± 1.80[b]	0.00± 0.00[d]
IL-11	6.82± 2.24	8.59± 3.56	4.07± 0.62	3.73± 0.42	3.19± 1.29	2.73± 1.48	2.82± 0.89	8.66± 5.09	2.36± 1.11	0.21± 0.13	8.12± 0.69[b]	9.88± 1.73[c]
IL-12p40	23.46± 4.83	5.40± 3.07	29.53± 8.39[b]	24.06± 7.53	8.86± 3.70	24.00± 7.92	18.87± 5.88	6.57± 3.01	22.33± 15.60	9.45± 4.13	42.18± 10.05[b]	29.98± 7.14
IL-12p70	11.32± 1.53	9.36± 1.83	14.89± 2.52	9.91± 3.34	14.87± 1.87	9.82± 2.55	13.09± 1.64	8.49± 4.08	19.64± 4.31	11.09± 0.96[a]	19.35± 2.22[b]	12.87± 1.69
IL-13	75.43± 7.45	31.03± 9.49[a]	56.86± 3.70	44.78± 8.21	67.41± 16.37	54.03± 10.79	62.08± 4.40	51.67± 4.62	75.47± 12.68	59.19± 6.33	105.3± 21.8[b]	76.58± 4.86
IL-15	84.94± 25.38	35.62± 12.81[a]	64.65± 7.48	54.45± 8.65	56.74± 4.95	55.36± 6.74	80.84± 29.77	72.16± 13.91	43.37± 10.50	59.57± 10.94	142.6± 24.0[b]	88.44± 7.93[d]
IL-17	0.84± 0.24	0.23± 0.16[a]	0.55± 0.14	0.58± 0.15	0.35± 0.18	0.98± 0.29[a]	0.81± 0.20	1.10± 0.26	0.51± 0.13	0.47± 0.08	1.26± 0.09[b]	0.66± 0.24[d]
IL-20	95.36± 22.73	213.78 ±7.07[a]	189.64 ±26.02	111.5± 22.6[c,d]	120.66 ±24.60	106.85 ±38.33	142.50 ±4.69	66.03± 18.94[d]	51.08± 2.63	67.76± 13.04	179.9± 34.1[b]	141.89 ±55.11
TNFα	0.00± 0.00	0.00± 0.00	0.63± 0.63	0.39± 0.39	0.63± 0.63	2.85± 1.18	1.39± 0.82	3.14± 0.68	1.79± 1.79	0.51± 0.38	1.76± 0.85	2.97± 1.23
VEGF-A	0.44± 0.03	0.52± 0.06	0.51± 0.07	0.94± 0.39[c,d]	0.52± 0.04	0.51± 0.03	0.49± 0.05	0.55± 0.03	0.58± 0.08	0.50± 0.05	0.69± 0.08	0.87± 0.15
EPO	248.90 ±36.79	212.09 ±30.37	243.55 ±82.59	105.92 ±39.26	275.40 ±68.11	311.22 ±46.91	443.80 ±63.20	400.51 ±83.54	76.00± 47.13	1553.3 ±86.5[a]	2137± 325[b]	2443± 463[c]
LIF	0.74± 0.41	0.31± 0.27	0.28± 0.11	0.08± 0.07	0.21± 0.08	0.02± 0.02	0.56± 0.27	0.27± 0.10	0.36± 0.17	0.16± 0.09	1.32± 0.39[b]	0.42± 0.13[d]

Data are expressed as mean ± SEM (N = 5 per group per time point). Concentrations are reported in pg/mL, except for G-CSF*, which is presented as Fluorescence Intensity because values exceeded the standard curve's upper limit.
Groups: S: Sham; V: Vehicle (PBS); TBI: 9.5 Gy total body irradiation; VT10: Vasculotide (10 μg/kg); VT20: Vasculotide (20 μg/kg).
Statistical Significance: $^{a}P < 0.05$ for S+V vs. TBI+V; $^{b}P < 0.05$ for TBI+V vs. TBI+VT10; $^{c}P < 0.05$ for TBI+V vs. TBI+VT20; $^{d}P < 0.05$ for TBI+VT10 vs. TBI+VT20. Data was analyzed via two-way ANOVA followed by Fisher's LSD multiple comparisons test.
