# Supplementary material for: Pre-Exposure Prophylaxis with Vasculotide Enhances Survival and Alleviates Hematopoietic and Gastrointestinal Injury Following Lethal Total Body Irradiation
Source: Int J Mol Sci. 2026 Feb 19;27(4):2001. doi: 10.3390/ijms27042001 (PMC12941350; doi:10.3390/ijms27042001)
Supplement: Supplementary file 1 [file ijms-27-02001-s001.zip › ijms-4129722 Supplemental Table S2 (final).pdf]

Supplemental Table S2. Multiplex analysis of serum chemokine profiles in mice on Days 1, 3, and 7 post-TBI.

	Day 1				Day 3				Day 7			
Chemokine	S+V	TBI+V	TBI+VT10	TBI+VT20	S+V	TBI+V	TBI+VT10	TBI+VT20	S+V	TBI+V	TBI+VT10	TBI+VT20
Eotaxin	460.7±165.2	356.6±104.6	578.51±68.69	531.82±97.27	551.9±176.1	799.01±71.88	922.80±90.80	917.17±79.59	564.65±44.07	847.1±133.0	1259±226[b]	863.4±64.8[d]
IP-10	42.59±3.30	71.60±15.51[a]	96.21±5.90[b]	88.03±4.40	41.17±4.01	87.40±6.76[a]	67.54±4.20[b]	81.54±6.67	46.88±2.51	58.31±4.02	70.81±8.03	51.96±2.36[d]
KC	20.39±8.14	34.04±9.40	56.26±9.30	63.14±30.06	18.51±5.47	122.2±47.6[a]	104.46±21.24	95.60±24.10	39.61±16.16	102.03±20.59	243.8±79.7[b]	105.2±22.8[d]
LIX	434.07±92.42	431.5±105.2	488.33±63.68	426.55±58.64	782.7±344.5	492.1±107.4	619.08±85.20	745.2±87.0	584.1±256.7	301.13±61.22	512.96±76.61	512.64±77.69
MCP-1	5.45±2.23	21.02±3.47	15.63±7.40	17.23±4.89	3.35±3.35	150.7±30.0[a]	173.20±30.56	121.49±9.12[d]	12.48±7.68	16.86±4.38	82.56±10.10[b]	73.51±17.02[c]
MCP-5	82.58±12.20	367.0±35.4[a]	307.45±50.52	302.78±63.53	105.54±21.72	603.3±66.2[a]	654.48±85.79	611.88±86.99	102.54±18.73	297.3±60.5[a]	439.66±37.78	481.7±43.1[c]
MDC	192.83±22.03	152.76±23.96	158.82±18.37	142.59±21.91	308.37±90.90	180.4±23.4[a]	212.87±24.93	198.24±18.45	312.09±61.53	43.57±8.43[a]	93.71±17.47	79.32±13.84
MIG	156.27±27.22	118.60±17.14	133.55±8.35	161.20±21.40	219.40±56.90	242.43±12.24	171.44±30.72	230.93±45.36	128.92±6.87	172.49±30.49	174.65±22.23	168.85±16.17
MIP-1α	15.58±8.18	15.38±6.52	30.30±4.36	65.9±36.1[c,d]	7.60±5.78	45.29±7.37[a]	46.35±5.47	40.40±5.38	14.43±14.43	0.00±0.00	3.28±3.28	1.63±1.63
MIP-1β	45.53±12.86	30.43±10.00	68.45±4.11[b]	85.37±29.23[c]	39.03±13.20	106.70±4.51[a]	80.57±9.93	77.95±1.92	32.69±16.60	45.77±3.16	85.46±8.47[b]	61.73±6.80
MIP-2	38.52±10.50	38.37±11.90	44.26±14.03	64.08±26.33	55.75±20.91	74.59±30.59	38.34±12.38	70.30±24.43	89.27±30.41	53.18±9.43	70.92±14.63	89.25±23.64
MIP-3α	12.10±4.06	30.71±8.36	17.89±8.12	29.75±27.52	8.75±8.75	22.21±10.98	3.46±1.59	8.18±3.41	14.75±9.11	95.06±15.88[a]	130.97±26.37	123.31±47.15
MIP-3β	16.13±11.34	86.40±28.65	52.48±30.11	108.13±25.43	16.51±16.51	20.17±19.38	41.47±29.96	74.93±22.23	13.95±13.95	59.50±19.26	92.01±24.43	199.7±92.5[c,d]
RANTES	14.31±3.45	8.87±1.78	8.45±2.49	9.77±0.81	14.25±3.79	10.77±0.60	10.24±2.56	10.03±1.80	13.68±2.66	8.86±2.43	20.61±1.24[b]	14.06±2.74
TARC	24.77±2.67	22.08±2.68	25.69±2.66	17.35±2.32	59.95±26.13	37.30±3.29	43.57±3.06	50.49±5.22	37.47±6.34	22.28±2.21	34.19±3.39	39.00±2.75
6Ckine / Exodus 2	8600.1±734.9	10286±216	11631±897	8693±1484[d]	5908±1652	10936±738 [a]	10399±786	11601±537	10323±638	10842±1138	11047±530	11837±769
Fractalkine	134.19±4.95	148.87±10.86	132.35±20.42	112.3±20.8[c]	105.34±15.40	161.47±7.18[a]	169.52±10.61	175.67±8.79	123.65±2.99	187.2±15.5[a]	243.08±8.48[b]	263.2±10.4[c]
IL-16	1880.2±117.2	1181±143[a]	1439.7±191.8	1532.5±210.7	1404.6±267.1	890.0±92.8[a]	925.0±165.2	816.9±100.2	2147.8±259.8	647.9±26.8[a]	592.6±77.0	781.3±130.2

Data are expressed as mean ± SEM (N = 5 per group per time point). Concentrations are reported in pg/mL.

Groups: S: Sham; V: Vehicle (PBS); TBI: 9.5 Gy total body irradiation; VT10: Vasculotide (10 μg/kg); VT20: Vasculotide (20 μg/kg).

Statistical Significance: [a]P < 0.05 for S+V vs. TBI+V; [b]P < 0.05 for TBI+V vs. TBI+VT10; [c]P < 0.05 for TBI+V vs. TBI+VT20; [d]P < 0.05 for TBI+VT10 vs. TBI+VT20. Data was analyzed via one-way ANOVA followed by Fisher's LSD multiple comparisons test.
